# Changes in host gene expression patterns underpin responses of the coral *Stylophora pistillata* to nutrient stress

**DOI:** 10.1038/s41598-025-12130-3

**Published:** 2025-08-01

**Authors:** Tessa M. Page, Cecilia D’Angelo, Jörg Wiedenmann, Gavin L. Foster

**Affiliations:** School of Ocean and Earth Science, University of Southampton, National Oceanography Centre Southampton, Southampton, SO14 3ZH UK

**Keywords:** Coral reefs, Transcriptomics, Nutrient stress, Phosphate limitation, Coral, Gene expression, Marine biology, Transcriptomics

## Abstract

The availability and stoichiometry of dissolved nutrients are known to have a significant effect on coral growth, biomineralisation, and stress tolerance. However, previous mechanistic studies have focused primarily on the photosynthetic symbionts. Here we studied the physiological and transcriptomic responses of the coral *Stylophora pistillata* exposed to four different concentration combinations of dissolved inorganic nitrogen (N) and phosphorus (P) over a period of eight weeks. Despite no significant effects on coral growth or calcification, corals from low P conditions bleached and had significantly fewer symbionts than those from high-phosphate treatments. Low P concentrations induced upregulation of ion transmembrane transporter activity, and downregulation of transcripts involved in phospholipid biosynthesis, protein processing, and protein maturation. Further, all enriched biological processes were related to phosphate metabolism. Our results suggest that *S. pistillata* controls a variety of molecular pathways to counteract the negative effects of insufficient nutrient supply.

**Supplementary Information:**

The online version contains supplementary material available at 10.1038/s41598-025-12130-3.

## Introduction

Stony corals support the structure and functioning of shallow water tropical coral reefs by building skeletons made of the aragonite polymorph of calcium carbonate (CaCO_3_)^1–3^through a process known as biomineralisation. Consequently, corals are vital ecosystem engineers, providing the majority of the three-dimensional structure to coral reef ecosystems, which in turn supports millions of marine species globally^4,5^. The growth, energy budget, and construction of their aragonite skeleton in many reef building corals is aided by the symbiotic relationship they maintain with dinoflagellate symbionts belonging to the family Symbiodiniaceae^6^. These symbionts within the coral host tissue facilitate the uptake and assimilation of dissolved inorganic nutrients including nitrate (NO_3_^−^) and phosphate (PO_4_^3−^)^7,8^, which are crucial for the biological and physiological functioning of corals and, by extension, the reefs they construct^9^.

Nutrient levels in waters surrounding coral reefs are typically relatively low, with concentrations below 1 µmol L^−1^ and 0.30 µmol L^−1^ for dissolved inorganic nitrogen and phosphorus, respectively^10,11^. Anthropogenic activities^9,12,13^ and natural processes^14–16^ (*for review* Ferrier-Pagés et al., 2000^17^) drive changes in the nutrient contents of the waters that bathe coral reefs, promoting variable responses, particularly via the enrichment of nitrogen and/or phosphorus^18,19^. Natural biological and oceanographic nutrient enrichment processes can render dissolved inorganic N or P locally or temporarily the most significant sources of N or P to symbiotic corals^20^with nitrate reaching concentrations of up to 11.5 µM in reef waters close to seabird colonies^16^. Extended exposure to limited nutrient availability and altered nutrient stoichiometry can affect the linear extension, growth, and overall skeleton building of corals, potentially making them more prone to bleaching, breakage, and other negative impacts from environmental stressors^18,21,22^. Species-specific differences may exist, for instance, growth of *Stylophora pistillata* declined with phosphorus enrichment^17^. In contrast, increased phosphate (+ 0.11–0.41 mg L^−1^) concentrations accelerated the growth of *Acropora murica*^23^. When both NO_3_^−^ and PO_4_^3−^ concentration were depleted, a broad range of species exhibited lower linear growth^18,20,24^. Similarly, lower linear extension and calcification, along with higher skeletal density and lower porosity, have also been observed under depleted nutrient or high N: P ratio conditions^18^. Balanced concentrations of PO_4_^3−^ and NO_3_^−^ enrichment following the Redfield stoichiometry for N: P^25^ of 16:1, however, led to faster linear skeletal extension, resulting in thinner, more porous, and less dense skeletons^18^.

Imbalanced ratios and limited availability of dissolved inorganic N and P have been found to rapidly reduce symbiont number in < 60 days^20^with a reduction in photosynthetic efficiency of the symbiont after just 14 days^22^. Furthermore, low availability of dissolved inorganic nutrients results in changes in the symbiont ultrastructure^21,26^with phosphate starvation causing the substitution of phospholipids for sulpholipids in symbionts and associated malfunctioning of photosynthesis^21,22^. Importantly, the latter was shown to have negative implications for heat and light stress tolerance^22^. Despite these rapid impacts on the symbionts, the coral host is able to maintain growth and calcification for up to ~ 7 weeks under continuous nutrient, nitrate and phosphate, limitation even without access to particulate food^20^. During this period, it has recently been shown that the host acquires nutrients through the continued digestion of the symbionts until their population is strongly depleted and the corals appear bleached^20^. This mechanism allows the holobiont to survive short-term disturbances in dissolved nutrient environment that can affect many coral reef systems around the world^9^. The reliance on the consumption of their vital symbionts can be considered an emergency response to insufficient nutrient supply that represents a major stress for the coral host. At the moment, the molecular basis of the response of the coral host to low nutrient stress is unclear.

As with many organisms, in corals several fitness-related trade-offs in response to stressful environments have been previously documented^27–30^. For instance, corals under stress may prioritise survival^27^biomass accumulation and energy storage^31^or photoprotection^30^ over (skeletal) growth. At the transcriptome level, stress responses often involve upregulation of genes related to chaperone activity and phosphorylation, while downregulating ribosome and mRNA processing genes^32^. Transcriptomic responses can also precede other physiological or biological changes^33,34^. For example, gene expression shifts induced by thermal stress in corals have been identified before bleaching occurs^33^thereby offering an early warning signal that can be exploited by conservation efforts^35^.

To build a deeper mechanistic understanding of the impact of nutrient availability and stoichiometry on coral host responses and physiological maintenance, we studied changes in the gene expression of *Stylophora pistillata* coral when provided with defined amounts of NO_3_^-^ and PO_4_^3–20^. The model coral *S. pistillata* was chosen for this experiment due to the availability of genome data^36–38^ and because of its known responses to low nutrient stress^20^. Specifically, our aim was to investigate gene expression and examine the initial responses of the coral host to the early phase of nutrient stress prior to any significant differences in growth or calcification. Throughout the 58-day study, we monitored symbiont health and function through zooxanthellae concentration and photosynthetic efficiency, as well as coral host metrics such as growth and calcification. In order to identify the genes and processes that play a role in how the coral host responds to changes in their nutrient environment, we then conducted differential gene expression analysis on the coral hosts to identify and quantify gene changes in response to nutrient treatments.

## Materials and methods

### Experimental design

Coral fragments (*n* = 36, ~ 2.5 cm in length) of *Stylophora pistallata* were removed from mother colonies grown in a ~ 2000 L experimental coral mesocosm system maintained by the Coral Reef Laboratory at the University of Southampton^39,40^. After fragmentation, 36 fragments were suspended on fishing line ~ 11 cm below the water surface and allowed to recover in a tank system with elevated nutrient levels (NO^3−^ ~ 15 µM; PO_4_^3−^ ~ 5 µM) for 4 months. Temperature, maintained with titanium stick heaters (Titanium Heater, D-D The Aquarium Solution Ltd, UK) attached to a controller (Dual Heating & Cooling Controller, D-D The Aquarium Solution Ltd, UK), was held at 26 ºC (± 0.2). Light, 12 h light: dark cycle, was provided from LED overhead lamps (Reef Pulsar, TMC, UK) at an intensity of ~ 150 µmol quanta m^−2^s^−1^ at the depth of coral.

Following acclimatisation, the 36 coral fragments were moved into a 614 L recirculating deplete nutrient system (NO^3−^: ~ 0.15 µM PO_4_^3−^: not detectable µM, method detection limit = 0.21 µM). Corals were hung across 4 experimental tanks (55 L each) fed from the larger, 614 L system. Temperature, salinity, and pH were continuously monitored in each tank through an Apex System (Apex A3, Neptune Systems, Netherlands). Temperature was controlled through titanium stick heaters attached to a controller (described above) and kept at 26 ºC (± 0.3). Salinity was maintained at 34.5 ± 0.61 psu. Light was maintained at 150 ± 15 µmol quanta m^−2^s^−1^ at depth of coral on a 12 h light: dark cycle. Circulation was provided within each tank by a wavemaker pump (Jecod Wavemaker Pump). Corals were moved daily across and within the 4 tanks to limit any differences in coral response due to position and tank. pH was measured twice daily using a portable pH meter (Mettler Toledo, SevenGo Duo SG98) paired with a pH electrode with integrated temperature probe (Mettler Toledo, InLab Pro) calibrated to the total scale (pH_T_) using Tris-HCl buffers^41^ across five temperatures ranging from 24 to 28 ºC. pH_T_ was 8.00 ± 0.05 throughout the duration of the experiment. Total alkalinity (*A*_T_) was measured every other day for the first 3 weeks of the experiment, and then every other week for the remainder of the experiment using open-cell potentiometric titrations (Apollo SciTech) following standard practices 3b^41^. Dissolved inorganic carbon was also measured weekly during the initial 4 weeks of the experiment, and then every 2–3 weeks following. pH_T_, *A*_T_, temperature, and salinity were used to calculate the remaining carbonate chemistry parameters using the Seacarb package version 3.3.1^42^ in the statistical computing program R, version 4.2.2 (Table S1). A 40 L water change was preformed daily and 4–8 g of reef foundation ABC+ (RedSea^®^) were added.

After corals were moved into the nutrient deplete system, defined nutrient pulses were administered immediately and over the following 58 days, as previously described^20^. Corals were exposed to one of the following four nutrient treatments for 3 h day^−1^ on five days per week: (1) a replete nutrient treatment (HNHP, NO^3−^: ~4.5 µM; PO_4_^3−^: ~0.3 µM; N:P = ~ 15); (2) a deplete nutrient treatment (LNLP, NO^3−^: not detectable; PO_4_^3−^: not detectable); (3) a skewed stoichiometry, high nitrate, low-phosphate treatment (HNLP, NO^3−^: ~8.0 µM; PO_4_^3−^: not detectable); and (4) an skewed stoichiometry low nitrate, high-phosphate treatment (LNHP, NO^3−^: not detectable; PO_4_^3−^: ~0.5 µM). Nutrient stoichiometry was chosen to follow Redfield stoichiometry (N: *P* = 16)^25^, in the replete, balanced treatment. Skewed N: P treatments deviated from the standard Redfield ratio^21^. Nutrient concentrations were manipulated in 2.5 L tanks that were placed within the larger 55 L tanks of the deplete nutrient holding system for the time of the treatment. Treatment tanks were run in triplicate with 2 or 3 coral fragments suspended within each 2.5 L tank. Each treatment tank served as a technical replicate, where nutrient treatments were mixed and controlled within each tank. Nutrient concentrations were monitored through colorimetric detection methods (DR900, Hach^®^), described in Rosset et al.^21^ throughout the 3 h pulses and specified nutrient levels were maintained throughout the pulse period by spiking with additional nutrient standards at 1.5 h to maintain intended nutrient treatment levels. Each treatment tank was equipped with an air stone connected to an air source to provide circulation during the pulse treatments. Care was taken so no water from the experimental treatment tanks entered the holding system. After each treatment, the treatment tanks were removed so that the corals experienced all the same recirculated water of the holding system. pH and temperature were monitored throughout and were maintained at 8.01 ± 0.02 and 26 ºC ± 0.02 respectively.

#### Symbiont photosynthetic efficiency and density

Maximum quantum efficiency of photosystem II (Fv/Fm) of zooxanthellae was measured twice a week using a submersible pulse amplitude modulated fluorometer (Diving-PAM, Walz, Germany) after 11 h dark acclimation with minimal background light levels. At the end of the experiment, symbionts were separated from host tissue and then counted with a haemocytometer to determine density^43^.

#### Growth and calcification

Measurements for growth were taken weekly. Linear extension and total area of all cultured corals were determined using standardized digital photography (Olympus TG-4) following published protocols^20^.

Coral skeletal dry weight was determined using the buoyant weight technique^44,45^. Buoyant weight measurements were made weekly. A Mettler Toledo XPR204 Analytical Balance outfitted with a weigh-below-hook was used to weigh corals. Temperature was kept constant at 26 ºC throughout weighing and salinity was recorded periodically. Seawater temperature and salinity were used to calculate seawater density and an aragonite density of 2.93 g cm^3^ was used to calculate skeletal dry weight. Skeletal dry weight was then used to estimate calcification rate.

#### Total RNA extraction and sequencing

Corals were sampled after 58 days in the nutrient treatments. Corals were removed from experimental nutrient tanks around midday and quickly flash frozen in liquid N_2_. Frozen corals were then transferred and stored at −80 ºC until further processing.

Approximately 75 mg of frozen tissue was removed from each sample and placed into 700 µl of lysis solution provided in the RNAqueous™ Total RNA Isolation Kit (Invitrogen™). Samples were homogenised in the lysis solution and then RNA was extracted from the homogenate following the RNAqueous™ Total RNA Isolation Kit (Invitrogen™) manufacturer’s protocol. RNA was resuspended in 50 µl of DNAse/RNAse-free distilled water. All RNA samples were cleared of potential DNA contamination using Invitrogen TURBO DNA-*free*™ Kit. Total RNA quantity and quality were tested using Qubit^®^ (Invitrogen) and Bioanalyzer (Agilent Technologies), respectively. Samples (*n* = 32) were sent to Novogene (Cambridge, UK) for Illumina stranded mRNA library preparation using polyA enrichment and sequencing. Libraries were sequenced using 150 bp paired-end reads on an Illumina NovaSeq 6000 System. Raw reads were de-multiplexed, trimmed, and adapters removed prior to releasing data. On average, total reads were 38 ± 1 million reads per sample (reported as mean ± standard error).

#### Bioinformatic analyses

Bioinformatic analyses were conducted on the University of Southampton’s High Performance Computer Cluster “Iridis”. Raw sequence reads were filtered to remove reads containing adapter contamination, those in which uncertain nucleotides (N) comprised more than 10% of either read, and those with over 50% of bases having a quality score below 5, following the approach from Yan et al.^46^. Quality control was performed on the resulting cleaned and trimmed sequence data using FastQC (*v* 0.12.0 Babraham Bioinformatics). Reads were aligned to the *S. pistillata* host genome assembly^38^ using HISAT2 (*v* 2.2.1)^47^ in the stranded paired-end mode and assembled using StringTie (*v* 2.2.1)^48^. Mapping precision of the generated, merged GFF files to the *S. pistillata* reference assembly was assessed using GFFcompare (*v* 0.12.6)^49^. A gene count matrix was generated using the StringTie python script prepDE^48^. *S. pistillata* protein sequences were annotated using Diamond (*v* 2.1.8)^50^ Uniprot^51^ and eggNOG^52^. FastQ files have been deposited on NCBI at BioProject PRJNA1156634.

#### Gene expression analysis

The StringTie^48^ generated gene count matrix was imported into R (*v.* 4.2.2)^53^ and filtered to remove transcripts that had more than 50% of counts under 10, which removed roughly 20% of transcripts with very low mean expression. Filtered and edgeR^54^ normalised counts for each sample were initially analysed using permutational multivariate analysis of variance (PERMANOVA) to identify if experimental nutrient treatments (NO_3_^−^ and PO_4_^3−^) were affecting gene expression patterns in the coral fragments. PERMANOVAs were conducted using the adonis2 function within the R package vegan^55^ with 9,999 permutations and calculated with Euclidean distance. Gene expression analysis was performed using edgeR^54^. Differential expression was determined using quasi-likelihood F-tests with default settings and the parameter ‘robust = TRUE’ to identify genes that were outliers from the mean-NB dispersion trend. Pairwise comparisons were performed on treatments, and genes that exhibited positive or negative log-fold changes were identified. Significantly differentially expressed genes (DEGs) were identified based on a false discovery rate (FDR) cut-off of 5% calculated using Benjamini-Hochberg method^56^. Heatmaps of gene expression patterns were made using the package pheatmaps^57^.

### Enrichment analysis

Functional overrepresentation analysis of DEGs was performed in the Cytoscape^58^ plugin BiNGO^59^. The hypergeometric test of gene ontology (GO) category used were “biological process” and “molecular function” with the annotated genome of *S. pistillata* as background, and Benjamini-Hochberg FDR corrected *p*-value with a cutoff of 0.05 was used. REVIGO was used to summarise visualise GO terms^60^.

#### Statistical analysis

All statistical analyses on growth parameters (i.e., area, linear extension, changes in skeletal weight, and calcification) and symbiont related measurements were conducted using R (*v.* 4.2.2). Data were tested for normality through the Shapiro-Wilk test and graphical analyses, all data met normality. To test significance after 8 weeks, one-way ANOVAs were run. If significance was found, ANOVAs were followed by Tukey’s post hoc test for pairwise comparisons. Linear models were built to test for significance of linear regressions using the lm() function in R.

## Results

### Consequences of short-term nutrient treatments to the coral host and symbionts

**Fig. 1:**
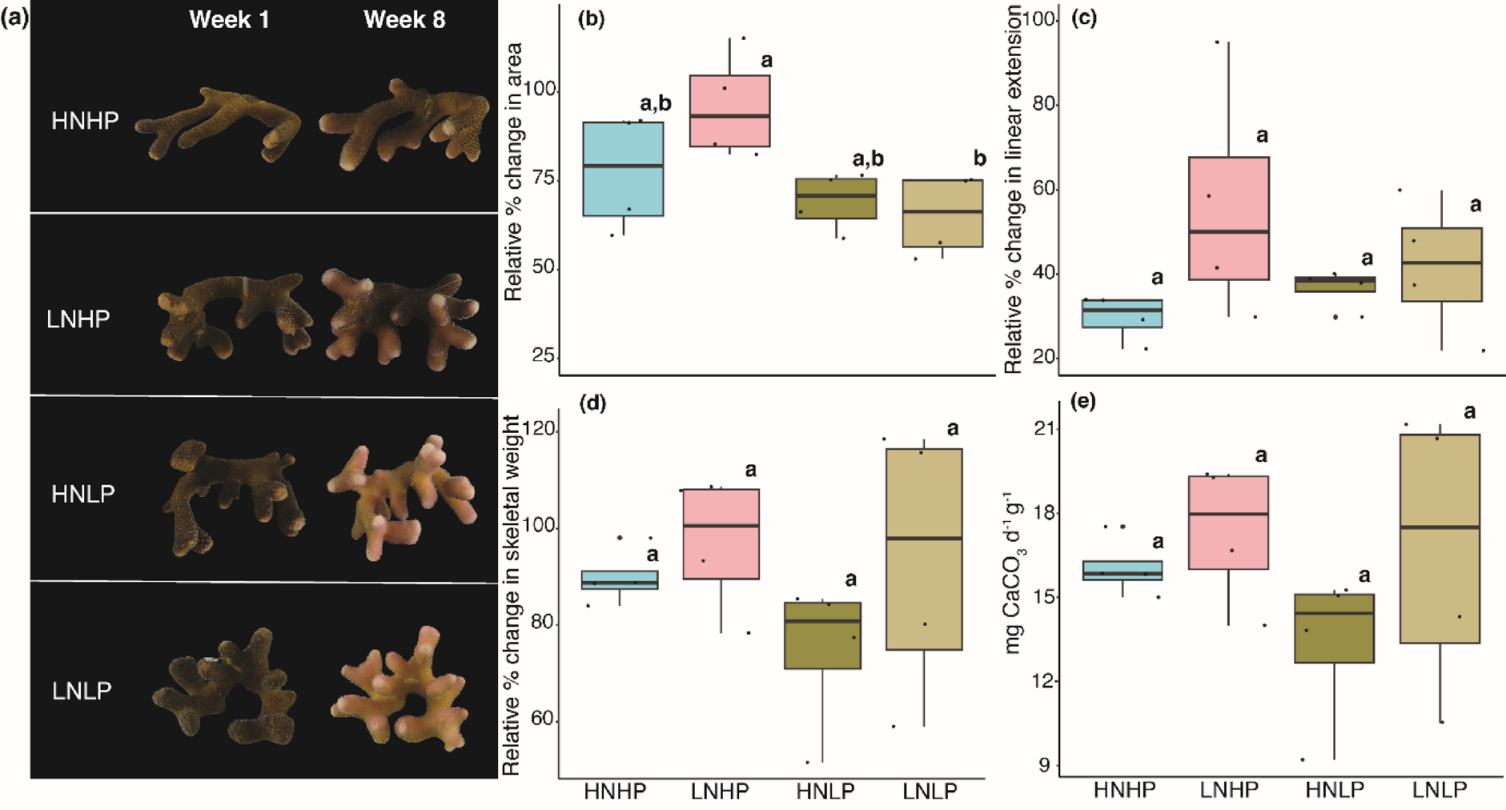
Effect of nutrient availability and stoichiometry on coral host physiology after 8 weeks (58 days) in treatment. (**a**) Representative fragments of *Stylophora pistillata* imaged through digital photography on day 1 of week 1, and at the end of the experiment (week 8). Colour loss is observed primarily in corals belonging to the LNLP and HNLP treatments. 8 replicate samples were analysed for each treatment. HNHP = high nitrate + high phosphate, LNHP = low nitrate + high phosphate, HNLP = high nitrate + low phosphate, and LNLP = low nitrate + low phosphate. (**b**,** c**,** d**,** e**) show relative percent change in average area, linear extension, skeletal weight (calculated from buoyant weight measurements of each coral fragment), and calcification rate as mg of CaCO_3_ d^−1^ g^−1^ for coral. Calcification rate was normalised to initial skeletal dry weight for each coral. Panels show measured physiological parameters as a function of nutrient treatment. Blue boxes correspond to HNHP (high nitrate + high phosphate), pink boxes correspond to LNHP (low nitrate + high phosphate), dark green boxes correspond to HNLP (high nitrate + low phosphate), and light green boxes correspond to LNLP (low nitrate + low phosphate). Lower case letters indicate significantly different treatments.

Photographs of the corals after 58 days (8 weeks) in nutrient treatments revealed that bleaching was restricted to the phosphate limited (LNLP, NO^3−^: not detectable & PO_4_^3−^: not detectable) and the phosphate starved (HNLP, NO^3−^: ~8.0 µM & PO_4_^3−^: not detectable) treatments (Fig. 1a). Growth of corals in all treatments was measured through changes in linear extension, area, and skeletal weight (Fig. 1), quantified through using standardised digital photography^20^ and the buoyant weight technique^44,45^. Although coral area was significantly impacted by treatment (ANOVA, *F*_*3,4*_ = 4.205, *p* < 0.05, Table S4, Fig. 1b), with corals from LNHP (NO_3_^−^: not detectable & PO_4_^3−^: ~0.5 µM) being slightly (< 40%) but significantly larger in area than corals from the LNLP treatment (Tukey, *p* < 0.05), no other skeleton growth-related parameter was significantly affected by nutrient treatment (Fig. 1, Table S5).

To analyse the density of symbionts (zooxanthellae) after 58 days in the nutrient treatments, symbionts were separated from the coral host tissue and then counted to determine density^43^. There was a significant effect of treatment (ANOVA, *F*_*3,4*_ = 6.073, *p* = 0.011) on zooxanthellae density in *S. pistillata* after 58 days (Table S2, Fig. 1a, Fig. S1a), with the zooxanthellae density of LNHP being > 60% higher than the zooxanthellae density of the HNLP and LNLP treatments (Tukey, *p* < 0.01 and *p* < 0.05, respectively). Despite these changes in symbiont density, there were no significant differences in photosynthetic efficiency, measured through maximum quantum efficiency of photosystem II (Fv/Fm) of zooxanthellae (ANOVA, *F*_*3,4*_ = 3.401, *p* > 0.05, Table S4, Fig. S1b). This suggests that although there were fewer symbionts, the functioning of their photosynthetic machinery was unaffected by the nutrient treatments.

### Differential transcriptomic response of the coral host is driven by phosphate

To compare differentially expressed genes across nutrient treatments, we analysed the transcriptomic profiles of the coral hosts via RNA sequencing (RNA-seq). We mapped our transcriptomic data to the publicly available genome of *S. pistillata*^38^. On average, 70% of the sequence reads mapped successfully to the reference genome of *S. pistillata*. 28,498 genes were generated for differential expression analysis. Transcriptomic data were initially assessed using a permutation multivariate analysis of variance (PERMANOVA) and partial least square discriminant analysis (PLS-DA) (Fig. S2 and Table 1). PERMANOVA run on the 28,498 genes showed the transcriptome-wide gene expression of host *S. pistillata* was significantly affected by the factor PO_4_^3−^ (present vs. absent PO_4_^3−^; PERMANOVA, *p <* 0.05, Table 1). Further, the PLS-DA showed clustering of treatments based on high or low PO_4_^3−^ concentration, with some treatments having greater within-group variability (Fig. S2). Following the finding of transcriptome-wide gene expression of the host *S. pistillata* to be significantly affected by PO_4_^3−^ independently, we focus hereon the effect of PO_4_^3−^. 

**Table 1:** Summary of results from the PERMANOVA test used to investigate the effects of nitrate (NO_3_^−^) and phosphate (PO_4_^3−^) on the transcriptome-wide expression of *Stylophora pistillata*..

Factor	df	SS	*R* ^2^	Pseudo-F	Pr (> F)
NO_3_^−^	1	1.4241e + 11	0.0660	2.2432	0.0989
PO_4_^3−^	1	1.9336e + 11	0.0897	3.0456	**0.0492**
NO_3_^−^: PO_4_^3−^	1	4.2527e + 10	0.0197	0.6699	0.5187
Residuals	28	1.7776e + 12	0.8245		
Total	31	2.1559e + 12	1.0000		

Text in bold indicate statistically significant differences (*p* ≤ 0.05).

**Fig. 2:**
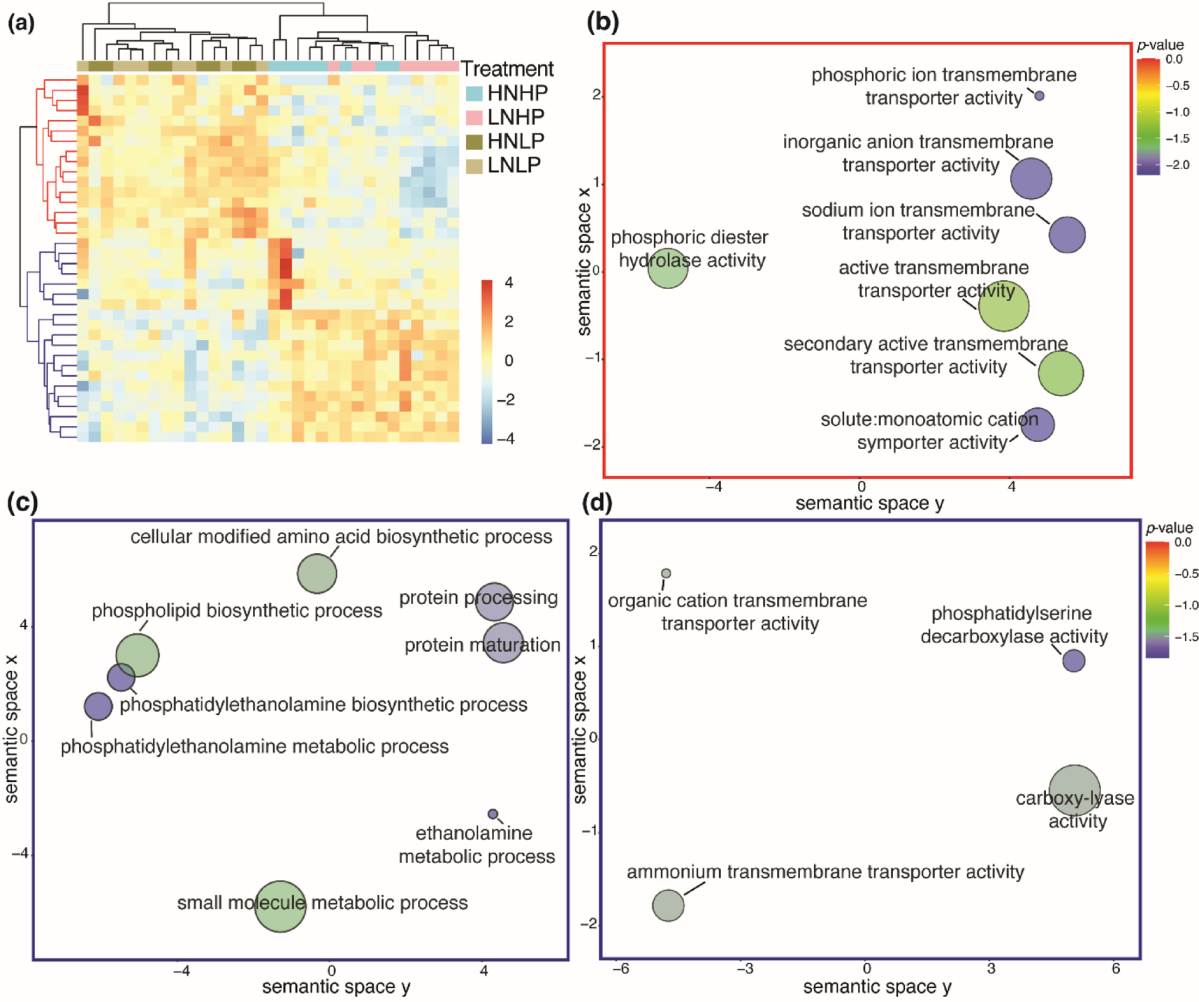
Nutrient treatments resulted in transcriptome modulation where clustering of up- and downregulated genes were apparent based on phosphate absence (HNLP and LNLP) or presence (HNHP and LNHP) in *Stylophora pistillata* after 8 weeks in treatment. (**a**) Heatmap of z-scored log2-counts per million (logCPM) values for differentially expressed genes (FDR < 0.05). Genes were identified as differentially expressed using edgeR’s^54^ generalised linear model framework with pairwise contrasts across all treatment combinations. Red indicates higher expression and blue indicates lower expression relative to the mean expression of each gene across all samples. (**b**) REVIGO visualisation of molecular function categories in differentially expressed genes (DEGs) belonging to the upregulated gene group from the low-phosphate treatments (HNLP and LNLP). (**c**) REVIGO visualisation of downregulated biological process categories in the low-phosphate treatments. (**d**) REVIGO visualisation of downregulated molecular function categories in the low-phosphate treatments. The axes in the plots have no intrinsic meaning, however, semantically similar gene ontological terms should be close together in a plot. The colour of the bubbles reflects the adjusted p-value obtained from the BiNGO enrichment analysis. The size of the spheres corresponds to the number of annotations for submitted gene ontological term IDs in the UniProt database, specifically a larger sphere indicates a more common GO term in the dataset. HNHP = high nitrate + high phosphate, LNHP = low nitrate + high phosphate, HNLP = high nitrate + low phosphate, and LNLP = low nitrate + low phosphate.

Using a differential gene expression analysis across the four nutrient treatment groups, we identified 36 significantly differentially expressed genes (DEGs; Bejamini-Hochberg corrected *p* < 0.05) linked to presence or absence of phosphate (HP or LP; Fig. 2), 16 transcripts were upregulated in the low-phosphate treatments (HNLP and LNLP) and 20 were downregulated (Fig. 2a; Table S3). Following differential expression analysis, gene ontology enrichment analysis was conducted on the DEGs. We found significantly enriched (*p* < 0.05, Hypergeometric Test with Benjamini-Hochberg’s False Discovery Rate correction) molecular function categories relating to transmembrane transport activity were upregulated in the phosphate limited treatments (Fig. 2b). Significantly enriched biological process categories that were downregulated in the low phosphate corals related primarily to metabolic and biosynthetic processes (Fig. 2c). Molecular function categories that were downregulated significantly in the low phosphate corals included carboxylase activity and ammonium transport activity (Fig. 2d).

**Fig. 3:**
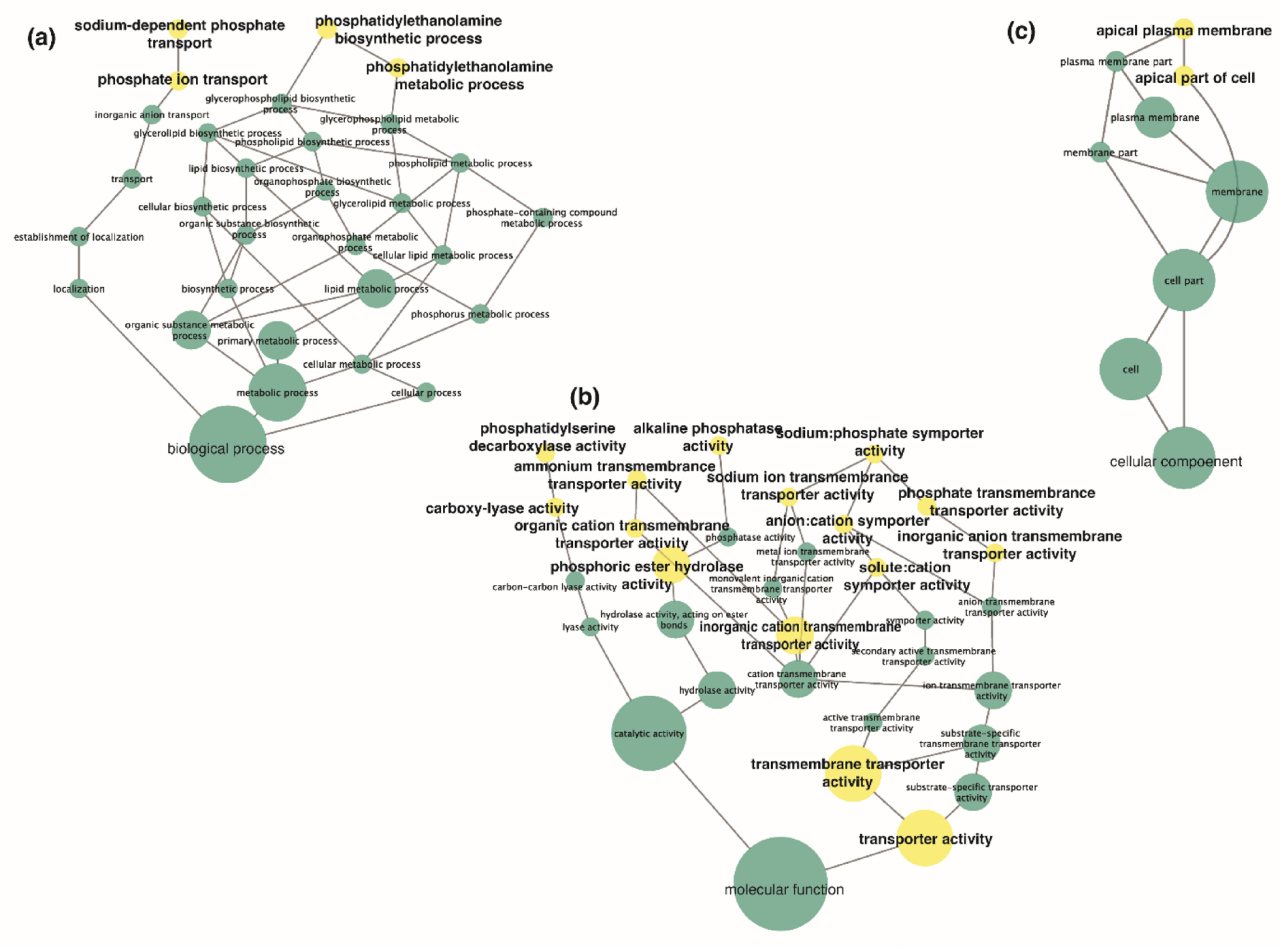
Network visualisation of enriched gene ontology (GO) categories within (**a**) biological processes, (**b**) molecular function, and (**c**) cellular component of differentially expressed genes (DEGs) in the coral host *Stylophora pistillata*. Network visualises entire transcriptome modulation (all DEGs) across all treatment groups. Each ellipse/node contains GO category that is enriched within the DEGs. Ellipses in yellow are significantly (*p* < 0.05, Hypergeometric Test with Benjamini-Hochberg’s False Discovery Rate correction) enriched (Table 2). The size of the node is related to the number of DEGs belonging to the GO category.

Enrichment analysis revealed significantly enriched GO categories (*p* < 0.05, Hypergeometric Test with Benjamini-Hochberg’s False Discovery Rate correction) for biological processes, molecular functions, and cellular components relating to transport of phosphate ion, inorganic cation, sodium ion, ammonium, and organic cation (Table 2). Enrichment analysis was viewed through network visualisation of biological processes, molecular functions, and cellular components for all DEGs (36) found in all treatment groups showing enrichment for global transcriptome modulation (Fig. 3). Although most GO descriptions only contained a single transcript, the GO descriptions containing 2 or more transcripts were inorganic cation transmembrane transporter activity, phosphoric ester hydrolase activity, transmembrane transporter activity, and transporter activity (Table 2; Fig. 3).

**Table 2:** Summary of significantly enriched gene ontology (GO) categories (*p* < 0.05, hypergeometric test with Benjamini-Hochberg’s false discovery rate correction) for biological processes, molecular functions, and cellular components. GO identification number, description, corrected *p*-value, and encoded protein accession IDs within each category are given.

GO ID	GO Description	Corrected *p*-val	Accession ID
Biological Process			
0044341	Sodium-dependent phosphate transport	3.20E-02	XP_022794503
0046337	Phosphatidylethanolamine metabolic process	3.20E-02	XP_022785767
0006817	Phosphate ion transport	3.20E-02	XP_022794503
0006646	Phosphatidylethanolamine biosynthetic process	3.20E-02	XP_022785767
Molecular function			
0022890	Inorganic cation transmembrane transporter activity	1.46E-02	XP_022793815 XP_022794503
0004609	Phosphatidylserine decarboxylase activity	1.46E-02	XP_022785767
0004035	Alkaline phosphatase activity	1.46E-02	XP_022794921
0015081	Sodium ion transmembrane transporter activity	1.46E-02	XP_022794503
0005436	Sodium: phosphate symporter activity	1.46E-02	XP_022794503
0015114	Phosphate transmembrane transporter activity	2.21E-02	XP_022794503
0015296	Anion: cation symporter activity	2.21E-02	XP_022794503
0015294	Solute: cation symporter activity	2.21E-02	XP_022794503
0015103	Inorganic anion transmembrane transporter activity	2.21E-02	XP_022794503
0042578	Phosphoric ester hydrolase activity	2.21E-02	XP_022794921 XP_022779363
0016831	Carboxy-lyase activity	4.64E-02	XP_022785767
0022857	Transmembrane transporter activity	4.68E-02	XP_022793815 XP_022794503 XP_022803467
0008519	Ammonium transmembrane transporter activity	4.68E-02	XP_022793815
0015101	Organic cation transmembrane transporter activity	4.68E-02	XP_022793815
0005215	Transporter activity	4.68E-02	XP_022793815 XP_022794503 XP_022803467
Cellular component			
0016324	Apical plasma membrane	3.35E-02	XP_022794503
0045177	Apical part of cell	3.35E-02	XP_022794503

## Discussion

Healthy corals obtain the energy and nutrients needed to grow and calcify through the symbiotic relationship with their algal symbionts and from feeding on particulate or dissolved organic matter^7,61^. In the current study, *S. pistillata* were not fed during the experiment and were cultured in filtered seawater, therefore the coral host had minimized access to particulate food as source of organic N or P. Nevertheless, across all treatments, corals continued to grow and calcify at similar rates for the duration of the experiment (58 days; Fig. 1b-e). In line with previous studies, we found that P limitation (LNLP) and P starvation (HNLP) resulted in a loss of symbionts and a bleached appearance of the corals (Fig. 1a^20,21^;), confirming previous findings that the continued growth and calcification was sustained by the consumption of the symbionts^20^. Here, we present evidence that the early response of the coral host to low nutrient stress is underpinned by significant changes in gene expression patterns. Specifically, *S. pistillata* from low-phosphate treatments showed a pronounced restructuring of their transcriptomes in response to stress associated with low-levels of nutrients (Figs. 1, 2 and 3).

Corals from the low-phosphate treatments upregulated transcripts relating to ion transport, such as inorganic anion transmembrane transporters, sodium ion transmembrane transport, and phosphoric ion transmembrane transport. The upregulation of ion transport related genes in corals has also been observed in response to temperature stress^62^ and pH stress^63,64^. In some cases, the upregulation of ion transport related genes has been suggested to counteract the negative effects of lowered pH on the calcification process. For instances, in a symbiotic coral, *Pocillopora damicornis*, lowered pH (7.8 and 7.4 pH) induced an upregulation of transport related genes, which could aid in sustaining calcification^64^. In the deep sea coral *Lophelia pertusa* (synonymised with *Desmophyllum pertusa*), a non-symbiotic coral, calcification was maintained at low pH by the upregulation of ion transport but this was coincident with the downregulation of genes involved in metabolic processes^63^. The upregulation of phosphoric diester hydrolase activity and the enrichment of phosphoric ester hydrolase and alkaline phosphatase activities during phosphate limitation could point toward a metabolic shift in corals that enables the utilisation of dissolved organic phosphorus when inorganic phosphate is limited. Alkaline phosphatases have been found to be higher in bleached corals than in symbiotic colonies, suggesting the loss of symbionts, and corresponding reduction of inorganic phosphate uptake, may lead to the reliance of the coral host on organic phosphate^65^. In the current study, the upregulation of transcripts in *S. pistillata* may have contributed to the sustained growth and calcification levels in corals from low-phosphate treatments by partially compensating for the reduced P-availability to the symbionts. Both, transcriptomic restructuring, leading to molecular trade-offs (e.g., upregulation of ion transport with a downregulation of metabolic processes), and the consumption of symbionts are likely energetically costly emergency measures of the coral host employs to maintain crucial physiological processes in the absence of phosphate.

We found *S. pistillata* in the low-phosphate treatments downregulate transcripts relating to processes involving phospholipid biosynthesis, In zooxanthellae, high levels of dissolved inorganic nitrogen combined with low levels of phosphate result in a substitution of phospholipids with sulpholipids^22^. This substitution results in a malfunction in the photosynthetic apparatus of the symbiont and in turn increases the coral susceptibility to temperature- and light-induced bleaching^22,66^. The results of this study suggest that the coral host cells also experience changes to the phospholipid content, as indicated by the downregulation of phospholipid biosynthetic processes. This may be the consequence of the low P availability or a mitigation measure to minimise the use of phosphorus. Future research should be performed to determine whether this response affects the integrity and functionality of biological membranes in the coral host cells. Transcripts related to protein processing were also downregulated in coral exposed to low phosphate. Protein processing is essential for maintaining cell membrane structure during heat adaptation, with upregulation of these transcripts suggested to play a role in heat stress adaptation^67^. In a study examining the effects of severe heat stress on the Red Sea *S. pistillata*, the upregulation of a large cluster of genes relating to protein processing was attributed to the relative resistance of the Red Sea *S. pistillata* to bleaching and its relatively low mortality rates^68^. The downregulation of transcripts relating to protein processing in our study therefore provides a potential mechanism for previous findings that P-limited corals have increased susceptibility to thermal stress^21,22,69^.

In addition to the upregulation of phosphate and inorganic anion transporter activities, other transcripts relating to transport processes are downregulated. Specifically, transcripts relating to the molecular function categories such as organic cation and ammonium transporter activity were downregulated significantly in the phosphate-limited corals. In our low-phosphate treatments, ammonium transport activity was downregulated, suggesting that phosphate limited *S. pistillata* lack the ability to take advantage of inorganic nitrogen compounds, specifically ammonium, dissolved in the surrounding seawater^70^. This deficiency could negatively affect the synthesis of amino acids and proteins. Any change to processes such as the synthesis of proteins or protein maturation can indicate significant shifts in cellular homeostasis and metabolism^71^. *S. pistillata* from the low-phosphate treatments also downregulated transcripts relating to protein maturation, indicating a shift in protein metabolic condition that may lead to potentially malfunctioning proteins and altered protein activity^71^. As with protein processing, this would impact how *S. pistillata* manages other environmental stressors, such as temperature, light, pollutant exposure, and disease, where molecular chaperone protein activity, such as heat shock proteins, play a crucial role^72,73^.

Similar GO categories relating to ion transport that were enriched in the upregulated transcripts of *S. pistillata* when phosphate was limited, were previously found to be enriched in calicoblastic cells and mesenterial filaments in a settling polyp of *S. pistillata*^74^. This suggests they may be crucial for biomineralisation due to their presence during this stage of development when coral polyps are beginning to develop their skeleton^74^. Furthermore, previous studies have linked ion transport to calcification^1,62,63,75^. Although we did find upregulation in transcripts relating to ion transport in corals under limited phosphate treatments, we did not identify differential regulation of any transcripts that have previously been suggested to be part of the biomineralisation toolkit that play a role in skeleton production in *S. pistillata*, such as carbonic anhydrases, which are involved in carbon supply for calcification^76^and acid-rich proteins, which have been suggested to be involved in calcium carbonate precipitation^77^. Of the 36 biomineralisation proteins identified in the skeletal organic matrix in Drake et al.^78^. none were significantly differentially expressed across our nutrient treatments, although all were found in our transcriptome datasets (Table S8). Our study did not find any significant differences caused by nutrient stress (e.g., limited phosphate) in calcification rates after 8 weeks in treatment, however, certain genes and pathways possibly relating to biomineralisation were differentially regulated, for example the upregulation of phosphoric ion transport. Phosphate ions are known to play critical roles in controlling crystallisation pathways of amorphous calcium carbonate^79^. Coral skeletons form via amorphous calcium carbonate precursors^80^; therefore, if the transport of phosphate ions is impacted, although we see no change in overall calcification rates, differences in the skeleton of phosphate limited and phosphate available corals may be expected. On the other hand, P in high concentrations has been considered a crystal poison and could be responsible for reduced skeletal density and calcification^81,82^. At present, it is not clear through which mechanism the upregulation of sodium-dependent phosphate transport, as found in the current study, could have aided in maintaining calcification rates in corals. In corals under phosphate limitation, the upregulation of sodium-dependent phosphate transport could have increased the uptake of phosphate across the coral host membrane^83^.

The analysis presented here provides evidence of transcriptomic reshuffling before changes in coral growth and calcification occur in response to low P availability. In particular, we observed stress-mitigation responses in *S. pistillata* when phosphate was limited. This was evident from *S. pistillata* maintaining similar levels of growth and calcification across treatments and the differential regulation of transcripts, with upregulation of transcripts relating to transport of phosphate and inorganic anion ions. The observed bleaching of *S. pistillata* under low-nutrient stress in the current study is in line with measurements of this species under comparable conditions, indicating that *S. pistillata* consumes their symbionts for nutrient supply when P is limited^20^. This suggests the transcriptomic response of the coral host is an attempt to mitigate the stress experienced due to the phosphate undersupply. However, this compensation may come at a cost to membrane stability and cellular metabolism and homeostasis^67,71^which would impact the overall tolerance of the coral holobiont to thermal and other environmental stress. Previous studies have found that after 60 days in phosphate limitation (i.e., limited phosphate availability in combination with low or excess nitrate availability), the coral host significantly decreases in overall fitness, growth, and calcification^17,18,20,24^ (Fig. S3). Therefore, transcriptomic or gene expression changes we observe here can serve as early warning signs, along with coral bleaching, of coral hosts response to environmental and anthropogenic nutrient stress, potentially helping to develop novel reef monitoring and conservation approaches.

## Electronic supplementary material

Below is the link to the electronic supplementary material.

Supplementary Material 1

## Data Availability

All data used to construct the principal figures of this manuscript have been uploaded to pure.soton.ac.uk (10.5258/SOTON.D3238). All sequencing data and corresponding metadata were deposited to NCBI and can be found at BioProject: PRJNA1156634 “Changes in host gene expression patterns underpin responses of the coral Stylophora pistillata to nutrient stress”. All bioinformatic scripts used for analyses are accessible through Github (https://github.com/tessamp/Spistillata_nutrients). The data that support the findings of this study are available through the provided BioProject number, or can be made available from the corresponding author, TM Page, upon request.
