# Supplementary material for: Changes in host gene expression patterns underpin responses of the coral Stylophora pistillata to nutrient stress
Source: Sci Rep. 2025 Aug 1;15:28065. doi: 10.1038/s41598-025-12130-3 (PMC12316964; doi:10.1038/s41598-025-12130-3)
Supplement: Supplementary file 1 — Supplementary Material 1 [file 41598_2025_12130_MOESM1_ESM.pdf]

Supplemental Information for:

Changes in host gene expression patterns underpin responses of the coral *Stylophora pistillata* to nutrient stress

Tessa M. Page*, Cecilia D'Angelo, Jörg Wiedenmann, Gavin L. Foster

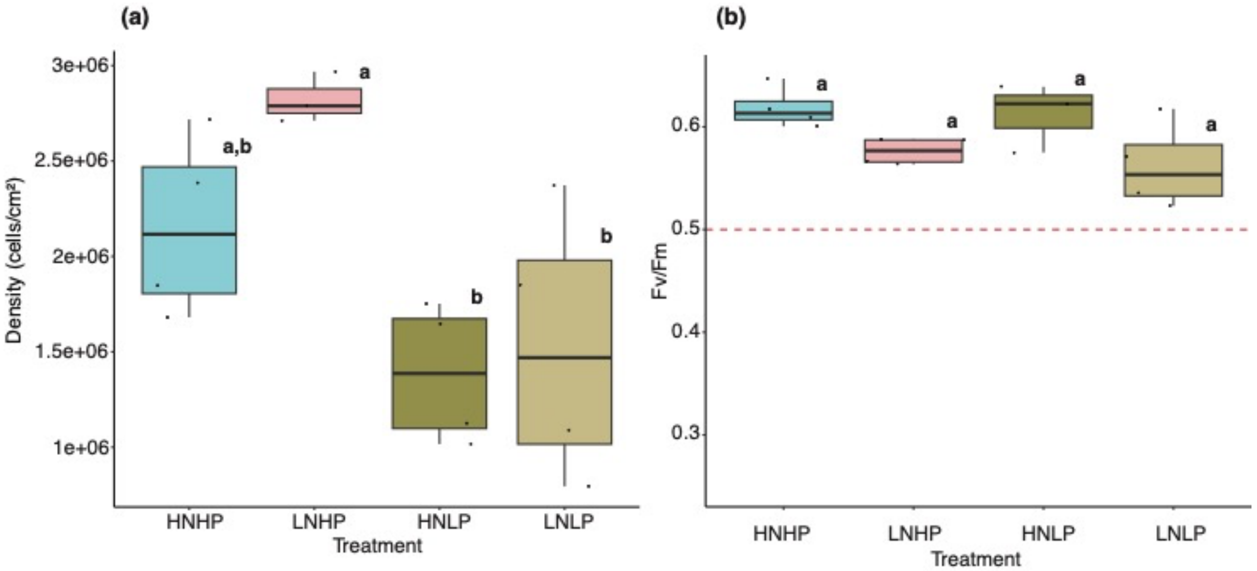

Figure S1. Effect of nutrient availability and stoichiometry on coral symbionts after 8 weeks (58 days) in treatment. (**a**) shows differences in zooxanthellae density (cells/cm^2) in *Stylophora pistillata* in desntiy of cells/cm^2. (**b**) shows differences in photosynthetic efficiency of zooxanthellae as quantum efficiency of photosystem II (Fv/Fm). The red dotted line shows the suggested cut-off threshold for the healthy efficiency of zooxanthellae. Panels show measured physiological parameters as a function of nutrient treatment. Blue boxes correspond to HNHP (high nitrate + high phosphate), pink boxes correspond to LNHP (low nitrate + high phosphate), dark green boxes correspond to HNLP (high nitrate + low phosphate), and light green boxes correspond to LNLP (low nitrate + low phosphate). Lower case letters indicate significantly different treatments.

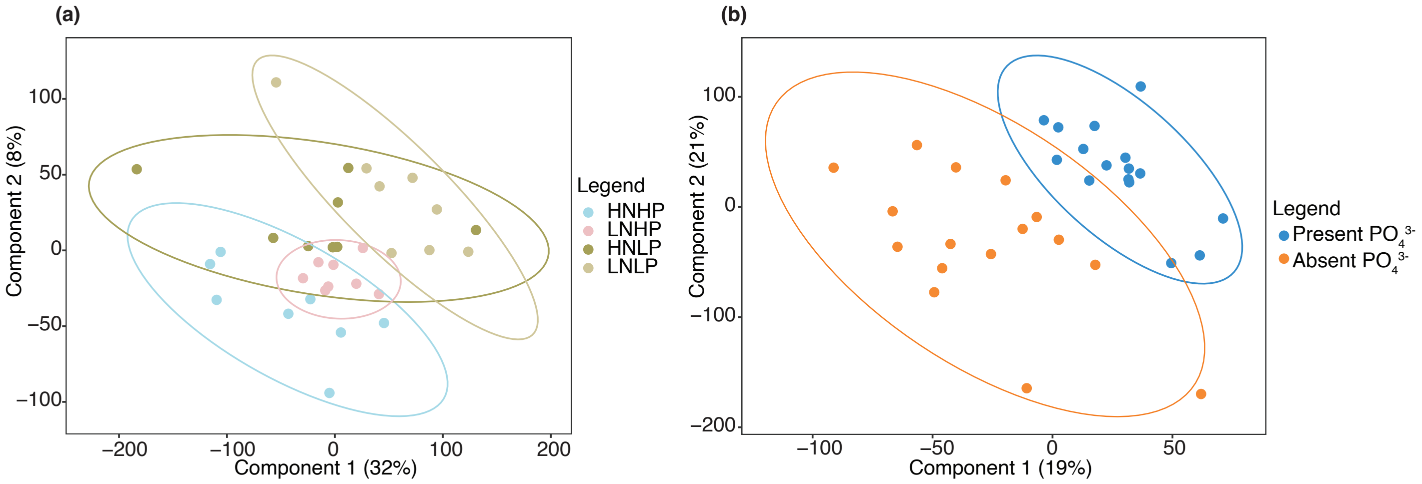

Figure S2. Partial least square discriminant analysis model comparing (**a**) the four nutrient pulse treatments of HNHP (high nitrate + high phosphate), LNHP (low nitrate + high phosphate), HNLP (high nitrate + low phosphate), and LNLP (low nitrate + low phosphate), and (**b**) comparing high and low PO_4^{3-} concentration in treatment. The amount of variance explained is shown on each axis in parentheses.

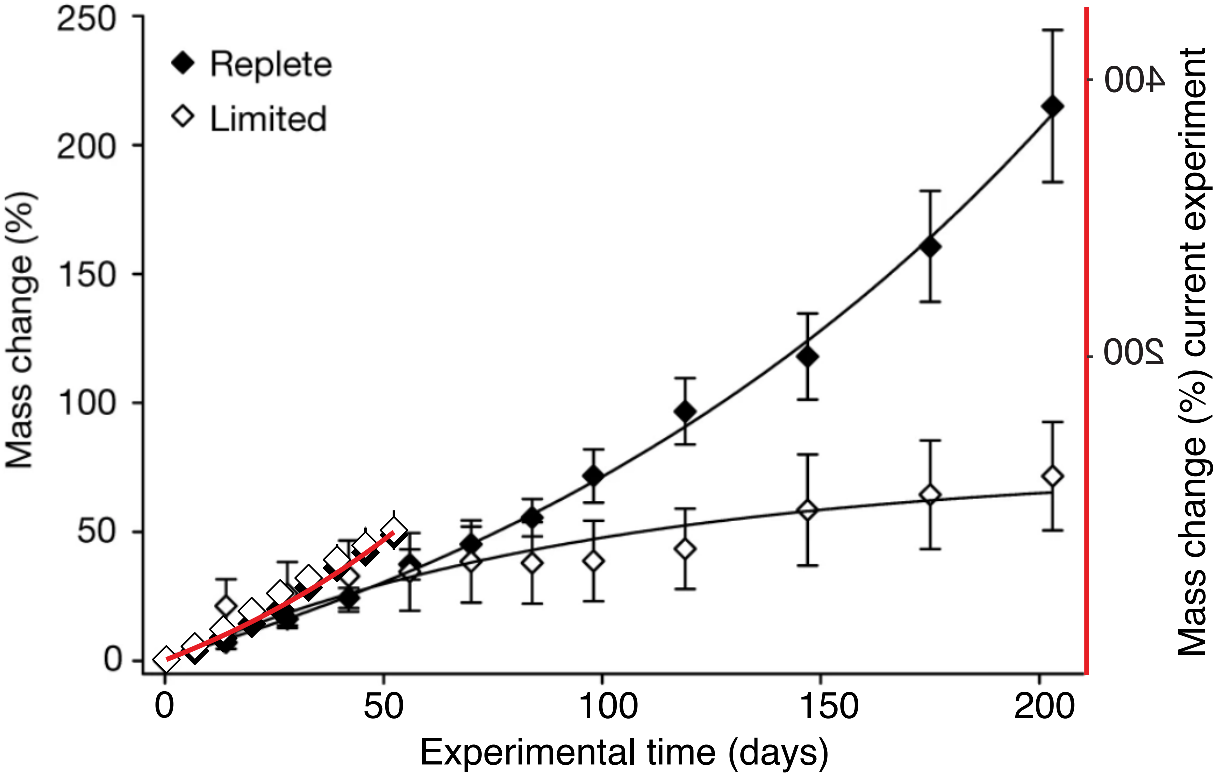

Figure S3. Figure adapted from Wiedenmann, *et al*. (Wiedenmann et al., 2023) showing changes in average coral mass (%) of ten different coral species. Graph shows coral mass in nutrient-limited and nutrient-replete conditions. Mass was recorded through taking the wet weight of coral fragments, see Wiedenmann, *et al*. (Wiedenmann et al., 2023) for further methods. Points on the left of the graph following the red trend line, ending ~50 days, are data from the current experiment overlayed. Data from the current experiment show mass change from skeletal weight measured through the buoyant weight technique (*33, 34*). Data from the current study shows only corals from the HNHP (high nitrate + high phosphate) and LNLP (low nitrate + low phosphate) conditions (similar to those described in Wiedenmann, *et al*. (Wiedenmann et al., 2023)).

Table S1. Mean composition of treatment tanks 1-4 culture water over 70 days of treatment. Values are reported as mean ± standard error.

	1	2	3	4
Salinity (g/kg)	34.65 ± 0.1	34.66 ± 0.1	34.68 ± 0.11	34.67 ± 0.1
T (ºC)	25.68 ± 0.02	25.71 ± 0.02	25.70 ± 0.02	25.70 ± 0.02
pH_T	8.04 ± 0.02	8.00 ± 0.01	8.01 ± 0.01	8.01 ± 0.01
*p*CO2 (µatm)	405.55 ± 14.06	433.21 ± 17.37	411.01 ± 10.24	423.42 ± 13.72
$[HCO_3^-]$ (µmol/kg)	1671.88 ± 18.47	1691.28 ± 13.35	1679.73 ± 11.06	1697.61 ± 13.44
$[CO_3^{2-}]$ (µmol/kg)	195.83 ± 4.48	187.20 ± 3.73	191.31 ± 2.71	190.94 ± 3.14
[DIC] (µmol/kg)	1879.01 ± 16.57	1890.55 ± 11.62	1882.48 ± 10.47	1900.35 ± 12.63
TA (µmol/kg)	2158.61 ± 14.37	2156.38 ± 9.78	2155.16 ± 10.06	2171.37 ± 11.77
$\Omega_{Aragonite}$	3.13 ± 0.07	2.99 ± 0.06	3.05 ± 0.04	3.05 ± 0.05
$\Omega_{Calcite}$	4.74 ± 0.11	4.53 ± 0.09	4.62 ± 0.07	4.62 ± 0.08

Table S2. Summaries of one-way ANOVAs for measured variables (i.e., zooxanthellae density, photosynthetic efficiency of the zooxanthellae, host area, host linear extension, host skeletal weight, and host calcification) in *Stylophora pistillata* exposed to different nutrient treatments after 8 weeks. Bolded values are bolded, followed by Tukey post hoc comparison.

	Term	df	sumsq	meansq	Statistic	p.value	Tukey
Zooxanthellae density	Treatment	3	4.43E+12	1.48E+12	6.073	**0.011**	LNHP > HNLP & LNLP
	Residuals	11	2.68E+12	2.43E+11			
Photosynthetic efficiency	Treatment	3	0.009	0.003	3.401	0.057	
	Residuals	11	0.009	0.001			
Area	Treatment	3	2235	745.1	4.205	**0.030**	LNHP > LNLP
	Residuals	12	2126	177.2			
Linear extension	Treatment	3	1509	502.9	1.798	0.201	
	Residuals	12	3356	279.7			
Skeletal weight	Treatment	3	10.72	3.573	0.755	0.541	
	Residuals	12	56.81	4.736			
Calcification	Treatment	3	39.98	12.33	1.171	0.361	
	Residuals	12	126.26	10.52			

Table S3. Results from differential gene expression analyses with EdgeR between four nutrient treatments after 58 days in treatment conditions.

Gene symbol	Annotation	logCPM	F	PValue	FDR
LOC111332081	fibroblast growth factor receptor 3-like	1.098	13.664	5.96E-06	0.018
LOC111323067	uncharacterized	1.692	12.260	1.75E-05	0.024
LOC111324915	matrix metalloproteinase-17-like	3.291	11.166	3.22E-05	0.033
LOC111324635	uncharacterized	2.758	11.320	2.71E-05	0.031
LOC111325774	uncharacterized	4.116	12.647	1.06E-05	0.021
LOC111333779	fibrillin-1-like	2.585	10.496	6.23E-05	0.045
LOC111324794	uncharacterized	5.150	13.401	8.20E-06	0.021
LOC111340820	uncharacterized	5.980	9.918	7.75E-05	0.049
LOC111322251	uncharacterized	5.794	11.282	3.02E-05	0.033
LOC111333226	Na-dependent phosphate transport	11.017	34.764	1.94E-10	0.000
LOC111329606	stress response protein NST1-like	5.487	11.599	2.21E-05	0.027
LOC111320895	glycerophosphodiester phosphodiesterase GDPD6-like	6.588	12.109	1.80E-05	0.024
LOC111325595	BUB3-interacting and GLEBS motif-containing protein ZNF207-like	7.155	10.597	4.62E-05	0.038
LOC111333580	alkaline phosphatase-like	9.147	10.035	7.35E-05	0.049
LOC111325835	roundabout homolog 1-like	4.037	14.948	2.31E-06	0.011
LOC111323003	phosphorylated carbohydrates phosphatase TM 1254-like	7.674	13.997	4.26E-06	0.016
LOC111336974	low-density lipoprotein receptor-related protein 8-like	8.096	12.107	1.54E-05	0.024
LOC111344578	membrane-associated progesterone receptor component 2-like	5.823	13.401	6.33E-06	0.018
LOC111337024	cystathionine gamma-synthase-like	6.816	11.047	3.30E-05	0.033

LOC111329978	arsenite methyltransferase-like	4.510	11.603	2.21E-05	0.027
LOC111337473	uncharacterized	6.723	10.519	4.90E-05	0.038
LOC111327308	uncharacterized	6.127	12.001	1.66E-05	0.024
LOC111334712	dual serine/threonine and tyrosine protein kinase-like	5.508	11.965	1.70E-05	0.024
LOC111333144	serine/threonine-protein phosphatase 1 regulatory subunit 10-like	6.056	20.987	7.39E-08	0.001
LOC111329415	RNA polymerase II subunit A C-terminal domain phosphatase SSU72-like	6.961	17.777	4.23E-07	0.002
LOC111341161	heat shock 70 kDa protein 12A-like	3.610	10.828	3.89E-05	0.035
LOC111319275	Down syndrome cell adhesion molecule-like protein Dscam2	5.247	10.563	4.74E-05	0.038
LOC111336140	uncharacterized	3.013	10.313	5.73E-05	0.042
LOC111326102	phosphatidylserine decarboxylase proenzyme, mitochondrial-like	7.385	23.864	1.78E-08	0.000
LOC111332672	putative ammonium transporter 1	8.536	12.844	9.22E-06	0.021
LOC111335987	von Willebrand factor A domain-containing protein 7-like	3.352	11.026	4.32E-05	0.037
LOC111330837	uncharacterized	5.293	11.386	3.72E-05	0.035
LOC111345582	uncharacterized	4.361	13.191	1.10E-05	0.021
LOC111341908	von Willebrand factor D and EGF domain-containing protein-like	4.908	11.282	4.00E-05	0.035
LOC111326564	uncharacterized	1.722	10.216	7.74E-05	0.049
LOC111320898	uncharacterized	3.216	10.577	6.54E-05	0.046

Table S4. 36 biomineralisation proteins identified in (Drake et al., 2013) and their presence or absence in our transcriptomic datasets. Gene identifier and accession number from (Drake et al., 2013). Transcript ID from our study is given. BLAST was used to identify similar transcript sequences in our transcriptomes using the sequences found for the 36 biomineralisation proteins in Drake *et al*. (2013).

Protein	Gene	Accession no.	Transcript ID
P1	g11108	KC509948	rna-XM_022929073.1
P2	g11187	KC493647	rna-XM_022926629.1
P3	g12510	KC342189	rna-XM_022927680.1
P4	g9861	KC342190	rna-XM_022941529.1
P5	g11674	KC150884	rna-XM_022927679.1
P6	g11666	KC149520	rna-XM_022939725.1
P7	g4601	KC342191	rna-XM_022939724.1
P8	g9654	KC342192	rna-XM_022931183.1
P9	g10811	KC000002	rna-XM_022929073.1
P10	g11107	KC509947	rna-XM_022929073.1
P11	g13727	KC342193	rna-XM_022924298.1
P123	g2385	JX891654	rna-XM_022941246.1
P13	g6918	KC342194	rna-XM_022945598.1
P14	g9951	KC342195	rna-XM_022927678.1
P15	g1532	KC493648	rna-XM_022924955.1
P16	g11702	KC342196	rna-XM_022924314.1
P17	g12472	KC149521	rna-XM_022943043.1
P18	g810	KC342197	rna-XM_022927695.1
P19	g20041	KC342198	rna-XM_022948277.1
P20	g6066	KC342199	rna-XM_022939001.1
P21	g18277	KC479163	rna-XM_022950591.1
P22	g19762	KC493649	rna-XM_022929805.1
P23	g1057	KC000004	rna-XM_022952349.1
P24	g15888	KC479164	rna-XM_022923985.1
P25	g11220	KC479165	rna-XM_022939185.1
P26	g1441	KC479166	rna-XM_022923985.1
P27	g18472	KC479167	rna-XM_022926180.1
P28	g11651	KC149519	rna-XM_022937687.1
P29	g13377	KC479168	rna-XM_022946048.1
P30	g11056	KC000003	rna-XM_022942523.1
P31	g20420	KC479169	rna-XM_022948277.1
P32	g5540	KC479170	rna-XM_022940136.1
P33	g8985	KC479171	rna-XM_022925629.1

P34	g1714	KC479172	rna-XM_022939001.1
P35	g7349	EU532164.1	rna-XM_022944179.1
P36	g13890	KC479173	rna-XM_022939001.1
